# Autologous Dental Pulp Stem Cell Transplantation for Mature Teeth With Apical Periodontitis and Root Perforation: Clinical and Radiographic Outcomes in Two Cases

**DOI:** 10.1155/crid/1311942

**Published:** 2026-07-10

**Authors:** Ryosuke Matsuki

**Affiliations:** ^1^ Matsuki Dental Clinic, Fukuoka, Japan

**Keywords:** apical periodontitis, cone-beam computed tomography, dental pulp stem cells, mineral trioxide aggregate, regenerative endodontic therapy, root perforation

## Abstract

**Introduction:**

This case report is aimed at evaluate the feasibility of regenerative endodontic therapy (RET) using autologous dental pulp stem cells (DPSCs) for previously endodontically treated, nonvital mature teeth with apical periodontitis and root perforation.

**Case Presentation:**

RET may offer an alternative to conventional retreatment for mature teeth with persistent apical periodontitis. Two patients aged 22 and 28 years were referred for treatment of maxillary anterior teeth. After mechanical enlargement and disinfection, a cervical root perforation in Case 1 was sealed with mineral trioxide aggregate. Autologous DPSCs isolated from extracted third molars were transplanted with granulocyte colony‐stimulating factor and atelocollagen into the disinfected root canals in both cases and additionally into the apical perforation site in Case 2. The tooth in Case 1 showed a positive response to electric pulp testing at 4 weeks after transplantation, whereas the tooth in Case 2 first showed positive responses to both electric pulp and cold testing at 12 weeks. Dental radiography and cone‐beam computed tomography demonstrated mineralized tissue formation in the apical part of the root canal and remission of the periapical lesions after 48 weeks. In Case 2, marked narrowing of the perforation site was also observed. These clinical and radiographic changes were further enhanced during follow‐up, reaching 96 weeks after transplantation. No local or systemic adverse events were observed in either patient throughout the observation period.

**Conclusion:**

In these two cases, RET using autologous DPSCs was feasible and safe, and was associated with favorable clinical and radiographic outcomes for up to 96 weeks. Because histological confirmation was not obtained and only two cases were included, these findings should be interpreted as preliminary evidence of feasibility rather than as proof of pulp–dentin complex regeneration and require validation in larger, controlled studies.

## 1. Introduction

Cell‐free regenerative endodontic therapy (RET) applied to mature teeth with apical periodontitis has shown clinically favorable outcomes, including the resolution of signs and symptoms and healing of apical periodontitis, comparable to traditional root canal treatments [1]. However, the tissue identified within the root canal system after RET comprises a combination of the periodontal ligament, cementum, and bone‐like tissue. This indicates that RET should be regarded as a process of repair and restoration of function rather than regeneration of the pulp–dentin complex [2]. Conversely, proper regeneration of the pulp–dentin complex in mature teeth with periapical lesions was reported in a recent preclinical study using RET harnessing autologous dental pulp stem cells (DPSCs) [3]. A regenerated pulp–dentin complex has the potential to fulfill immunological and sensory functions and contribute to the long‐term preservation of teeth by helping prevent the progression of caries, apical periodontitis, and further tooth fracture. Clinical trials and a case report have demonstrated the safety and efficacy of RET using autologous and allogeneic mesenchymal stem cells, including DPSCs, in mature teeth with apical periodontitis [4, 5]. However, further clinical evidence is required, particularly regarding the treatment of refractory apical periodontitis.

One potential complication of root canal preparation is root perforation, which occurs when there is communication between the root canal system and the external tooth surface. If not properly repaired, this condition can lead to inflammation, periodontal ligament deterioration, and eventual tooth loss [6]. Mineral trioxide aggregate (MTA) is one of the most widely used materials for repairing root perforations. This is attributed to its biocompatibility and release of a high concentration of calcium, which enhances the repair of periodontal tissues and long‐term stability [7]. A 10‐year follow‐up prospective cohort study reported the long‐term stability of MTA in the treatment of root perforations, with only 8% of the cases showing little or no healing [8]. However, the success rate of root perforation repair with MTA is lower in older root and furcal perforations [9]. Recently, a case report demonstrated the application of RET using allogeneic umbilical cord mesenchymal stem cells in a mature tooth with apical periodontitis and root perforation, showing remission of periapical lesions and positive sensory responses at 6 and 12 months postoperatively, with no adverse effects reported [10]. However, the follow‐up period was limited to 1 year, and neither long‐term clinical outcomes nor histological evaluations of the regenerated tissue were provided. Although allogeneic umbilical cord–derived mesenchymal stem cells are clinically available for certain indications, their use in RET requires further validation and indication‐specific approval, particularly regarding standardized quality, potency, and safety. The author is unaware of prior reports describing RET using autologous DPSCs for previously treated mature teeth with apical periodontitis and root perforation.

In a previously endodontically treated mature tooth presenting with both apical periodontitis and root perforation, clinical decision‐making is more complex than for either condition alone. Conventional alternatives include orthograde retreatment combined with MTA‐based perforation repair, surgical endodontic intervention (apicectomy with retrograde filling), and extraction followed by prosthetic or implant‐supported replacement. Each option has identifiable limitations in young patients with anterior teeth in the esthetic zone: Orthograde retreatment combined with MTA repair shows a reduced success rate when the perforation is aged or located near the periapical lesion [8, 9]; surgical approaches may compromise the limited buccal bone around maxillary incisors, particularly for cervical perforations; and extraction sacrifices the natural dentition and its proprioceptive function in young patients who may otherwise live with the replacement for several decades. None of these options re‐establish living tissue within the root canal, so the long‐term outcome depends on the durability of restorative materials rather than on biological integration. RET using autologous DPSCs was therefore considered as an exploratory tooth‐preserving option in the present two patients. The rationale for selecting this approach included (i) young age (22 and 28 years) and the correspondingly long expected functional service of the affected teeth; (ii) involvement of maxillary central incisors in the esthetic zone, where extraction‐based options carry esthetic and long‐term maintenance disadvantages; (iii) persistent apical periodontitis despite previous endodontic treatment, indicating that conventional orthograde retreatment alone was unlikely to be sufficient; (iv) coexistence of root perforation, which further reduces the predictability of MTA‐only repair, especially when the perforation is located near or apical to the lesion [8, 9]; and (v) the patients′ informed preference for a tooth‐preserving regenerative option after being fully advised of the experimental nature of the therapy and of all available alternatives. The therapy was provided within the regulatory framework of Class II regenerative medicine in Japan, with prior review by a Certified Committee for Regenerative Medicine and acceptance of the regenerative medicine provision plan by the Ministry of Health, Labour and Welfare.

Thus, this case report is aimed at providing preliminary clinical and radiographic evidence on the feasibility of RET using DPSCs in two complex cases involving mature teeth with apical periodontitis and accidental root perforation. In one case, the perforation was sealed with MTA before initiating the root canal treatment, whereas in the other case, the perforation was directly filled with DPSC suspensions concurrently with cell transplantation. These two cases therefore represent different therapeutic scenarios rather than equivalent interventions, and are presented as distinct clinical situations.

## 2. Case Presentation

This case report was prepared in accordance with the Preferred Reporting Items for Case reports in Endodontics (PRICE) 2020 guidelines and the CARE guidelines; a completed CARE checklist was submitted as a separate file. A concise clinical timeline for each case is provided in Table 1, and a summary comparison of baseline and follow‐up findings, including percussion, palpation, mobility, probing depth, electric pulp testing (EPT), cold testing, lesion size, and perforation characteristics, is provided in Table 2. Periapical bone status was additionally graded on cone‐beam computed tomography (CBCT) images using the cone‐beam computed tomography periapical index (CBCT‐PAI), which combines a six‐point ordinal scale (0–5) reflecting the largest dimension of the periapical radiolucency with two cortical bone modifiers (E, expansion; D, destruction), as proposed by Estrela et al. [11]. All procedures were conducted in accordance with the Declaration of Helsinki. The treatments were provided as Class II regenerative medicine under Japan′s Act on the Safety of Regenerative Medicine. The clinical protocol for autologous DPSC transplantation was reviewed by a Certified Committee for Regenerative Medicine (Certification Number NA8200004), and the regenerative medicine provision plan was accepted by the Japanese Ministry of Health, Labour and Welfare before treatment initiation (Provision Plan Number PB7210011). Written informed consent was obtained from all patients for the treatment and for publication of anonymized clinical data and accompanying images. Patient information was deidentified, and clinical records, follow‐up assessments, and adverse‐event monitoring were maintained in accordance with the accepted provision plan and applicable regulatory requirements. Although this report describes only two clinical cases, the favorable clinical course, safety profile, and multimodal follow‐up findings support the feasibility of autologous DPSC transplantation performed within a regulated Class II regenerative medicine framework. Although not confirmatory evidence of efficacy, these cases provide encouraging preliminary evidence to support future clinical investigations of RET using DPSCs in previously treated mature teeth with apical periodontitis and root perforation.

**Table 1:** CARE‐compliant clinical timeline of the two cases.

Phase/visit	Timepoint	Summary from the episode of care
Case 1—22‐year‐old male, left maxillary central incisor		
Pretreatment history	Age 15 (≈7 years before referral)	Traumatic injury to the left maxillary central incisor → pulp necrosis. Endodontic treatment performed at another clinic.
Initial visit	Age 22/Day 0 (reference for this case)	Referred for pulp regenerative cell therapy. Findings: periapical tenderness, cervical discoloration, EPT (−). Radiograph: Filling material extruded beyond the apex with periapical radiolucency. CBCT: periapical bone resorption and labial cervical perforation. Informed consent obtained.
Disinfection—Session 1	Day 28	Removal of gutta‐percha; cervical perforation curetted and sealed with MTA (TMR MTA cement; Yamakin, Konan, Japan). Sequential irrigation/medication protocol Steps 1–5 (Table S1) applied; 0.015% levofloxacin‐loaded nanobubbles as intracanal medication. PCR: positive.
Disinfection—Session 2	Day 89 (9 weeks after Session 1)	Asymptomatic; gingival recession had exposed the labial MTA to the oral environment. Doripenem (Finibax)–loaded nanobubbles based on *Streptococcus mitis* identified by sequencing. PCR: weak positive.
Disinfection—Session 3	Day 103 (2 weeks after Session 2)	Asymptomatic. Doripenem‐loaded nanobubbles continued. PCR: weak positive.
Third molar extraction	Day 123	Maxillary left third molar extracted and transported to the cell‐processing facility (Air Water Aeras Bio Inc., Kobe, Japan).
Cell‐processing phase	Day 124–Day 144 (≈20 days)	Autologous DPSCs isolated by enzymatic digestion, expanded to Passage 4, and cryopreserved. Release tests (identity, viability, doubling time, sterility, endotoxin, mycoplasma) all met predefined criteria (Table 2).
Disinfection—Session 4	Day 159 (8 weeks after Session 3)	PCR: positive; *Lautropia mirabilis* identified by sequencing. Antibiotic switched to ampicillin (Viccillin; Meiji Seika Pharma, Tokyo, Japan)–loaded nanobubbles.
Disinfection—Session 5	Day 173 (2 weeks after Session 4)	PCR: negative—aseptic conditions confirmed (173 days from initial visit; total of five disinfection sessions).
Cell transplantation	Day 193 from initial visit (= Tx Day 0)	Final irrigation: 17% EDTA (2.5 mL, 2 min) followed by sterile saline (5 mL). 2 × 10^5^ autologous DPSCs delivered with G‐CSF and atelocollagen into the root canal. Gelatin sponge (Spongel) placed at the level of the MTA‐sealed cervical perforation. Access cavity sealed with Biodentine (Septodont) and composite resin.
Follow‐up—Week 1	Tx + 1 week	Asymptomatic.
Follow‐up—Week 4	Tx + 4 weeks	Asymptomatic; EPT (+) first detected.
Follow‐up—Week 12	Tx + 12 weeks	Asymptomatic; EPT (+) sustained. Radiograph: previously extruded radiopaque material gradually migrating into the root canal space.
Follow‐up—Week 24	Tx + 24 weeks	Asymptomatic; EPT (+) sustained.
Follow‐up—Week 48	Tx + 48 weeks	Asymptomatic; EPT (+) but beginning to weaken. CBCT: resolution of apical lesion; significant mineralization in the apical part of the root canal.
Follow‐up—Week 96	Tx + 96 weeks	Tooth survived; asymptomatic. Apical calcification and lesion resolution more pronounced; no change in midroot dentin thickness. EPT response further diminished. No local or systemic adverse events throughout the 96‐week follow‐up.
Patient perspective	At final follow‐up	No subjective tooth‐related symptoms were reported, and the patient was satisfied with the regenerative endodontic treatment itself, although slight dissatisfaction with gingival recession remained.
Total observation period	Initial visit → Tx + 96 weeks (≈924 days)	No local or systemic adverse events were observed throughout the entire observation period.

Case 2—28‐year‐old female, right maxillary central incisor		
Pretreatment history (childhood)	Age 13 (≈15 years before referral)	Caries treatment of the right maxillary central incisor; subsequent spontaneous pain led to pulpectomy at another clinic.
Pretreatment history (early adulthood)	Early 20s (≈5 years before referral)	Onset of apical tenderness; root canal retreatment performed at another clinic, but symptoms persisted.
Initial visit	Age 28/Day 0 (reference for this case)	Referred during ongoing orthodontic treatment. Findings: persistent apical tenderness, cervical discoloration, EPT (−). Radiograph: sparse root canal filling. CBCT: periapical lesion and perforation near the root apex. Informed consent obtained.
Disinfection—session 1	Day 52	Removal of gutta‐percha. Apical perforation prepared under microscope and intentionally left unsealed (no MTA presealing). Sequential protocol Steps 1–5 (Table S1); 0.015% levofloxacin‐loaded nanobubbles as intracanal medication. PCR: positive.
Coronal sealing and internal bleaching phase	Day 135–Day 163 (≈4 weeks)	Upper part of the root canal sealed with hydraulic cement; sodium perborate placed in the coronal cavity for 4 weeks for internal bleaching of the discolored crown. Levofloxacin‐loaded nanobubble medication maintained in the apical portion.
Third molar extraction	Day 163	Patient perspective rows (highlighted) are required by the CARE guideline and remain to be completed.
Cell‐processing phase	Day 164–Day 185 (≈21 days)	Patient perspective rows (highlighted) are required by the CARE guideline and remain to be completed.
Disinfection—Session 2	Day 191 (≈20 weeks after session 1)	PCR: negative—aseptic conditions confirmed (191 days from initial visit; total of 2 disinfection sessions). Sequencing did not identify any pathogenic bacteria during the treatment course; levofloxacin was therefore used as the sole antibiotic throughout.
Cell transplantation	Day 226 from initial visit (= Tx Day 0; after completion of orthodontic treatment)	Final irrigation: 17% EDTA (2.5 mL, 2 min) followed by sterile saline (5 mL). 2 × 10^5^ autologous DPSCs delivered with G‐CSF and atelocollagen into the root canal AND directly into the apical perforation site (without prior MTA sealing). Collagen–hydroxyapatite scaffold (ReFit Dental; HOYA Technosurgical Corp., Tokyo, Japan) placed over the canal orifice. Access cavity sealed with Biodentine (Septodont) and composite resin.
Follow‐up—Week 1	Tx + 1 week	Asymptomatic.
Follow‐up—Week 4	Tx + 4 weeks	Asymptomatic; EPT (−) and cold test (−).
Follow‐up—Week 12	Tx + 12 weeks	Asymptomatic; positive responses to both EPT and cold testing first observed.
Follow‐up—Week 24	Tx + 24 weeks	Asymptomatic; EPT (+) and cold test (+) sustained. Progressive increase in intracanal radiopacity.
Follow‐up—Week 48	Tx + 48 weeks	Asymptomatic; EPT (+) and cold test (+) persisted. CBCT (sagittal/axial): regression of apical lesion and mineralized tissue formation at the perforation and apical sites.
Follow‐up—Week 96	Tx + 96 weeks	Tooth survived; asymptomatic. Near‐complete closure of the perforation site; further apical mineralization. 3D‐reconstructed CBCT (DenPre 3D Lab, Dental Prediction, Tokyo): selective calcification on the perforated side, suggesting selective repair of the perforation. No local or systemic adverse events.
Patient perspective	At final follow‐up	No subjective tooth‐related symptoms were reported, and the patient was satisfied with the regenerative endodontic treatment itself, although slight dissatisfaction with residual mild tooth discoloration remained.
Total observation period	Initial visit → Tx + 96 weeks (≈898 days)	No local or systemic adverse events were observed throughout the entire observation period.

*Note:* Plus and minus signs (+/−) denote positive/negative responses; (±) denotes weak positive. Day 0 of each case is defined as the initial visit; Tx Day 0 is the day of cell transplantation. Items marked “Day X” are case‐specific values to be filled in from clinical records.

Abbreviations: CBCT, cone‐beam computed tomography; DPSC, dental pulp stem cell; EDTA, ethylenediaminetetraacetic acid; EPT, electric pulp testing; G‐CSF, granulocyte colony‐stimulating factor; GP, gutta‐percha; MTA, mineral trioxide aggregate; nanobubbles (NB) used as the irrigant/medication carrier; NaOCl, sodium hypochlorite; PCR, polymerase chain reaction; Tx, transplantation.

**Table 2:** Patient profile, transplanted DPSC characteristics, clinical and radiographic timeline, and outcomes in the two cases.

	Case 1	Case 2
**Patient profile**		
Age, sex	22 years, male.	28 years, female
Affected tooth	Left maxillary central incisor.	Right maxillary central incisor
Relevant history	Trauma at age 15 → pulp necrosis → endodontic treatment.	Caries treatment at age 13 → pulpectomy at another clinic; persistent apical tenderness despite retreatment; orthodontic treatment ongoing
Chief complaint/EPT	Periapical tenderness, cervical discoloration/negative.	Persistent apical tenderness, cervical discoloration/negative
Initial radiograph/CBCT	Filling material extruded beyond apex with periapical radiolucency; periapical bone resorption and labial cervical perforation on CBCT.	Sparse root canal filling; periapical lesion and perforation near the root apex on CBCT
**Baseline clinical examinations**		
Percussion	None.	None
Palpation	None.	None
Mobility (Miller class)	Class 0.	Class 0
Probing depth (mm)	≦ 3.	≦ 3
**Transplanted DPSCs—quality and safety**		
Source tooth	Left maxillary third molar.	Left maxillary third molar
CD29/CD105/CD31 (%)	99.80/99.34/0.00.	99.02/98.42/0.00
Viability (%)/doubling time (h)	94.4/24.5.	95.7/13.7
Bacteria/endotoxin/mycoplasma	Negative/< 1.00 pg mL^−1^/negative.	Negative/< 1.00 pg mL^−1^/negative
**Root canal disinfection**		
Perforation management before disinfection	Cervical perforation sealed with MTA.	Apical perforation prepared under microscope; left unsealed
Antibiotic‐nanobubble regimen	Levofloxacin → doripenem → ampicillin (changed per identified pathogen).	Levofloxacin only
Pathogens identified by PCR/sequencing	*Streptococcus mitis* (1st), *Lautropia mirabilis* (4th).	No pathogenic bacteria identified
Sessions to PCR negativity	5.	2
**Cell transplantation**		
DPSC dose/carrier	2 × 10^5^ cells/G − CSF + atelocollagen.	2 × 10^5^ cells/G − CSF + atelocollagen
Scaffold over cells	Gelatin sponge (Spongel).	Collagen–hydroxyapatite (ReFit Dental)
Cells delivered into perforation	No (MTA‐sealed beforehand).	Yes—direct fill of apical perforation
Coronal seal	Biodentine + composite resin.	Biodentine + composite resin
Additional procedure	—	Sodium perborate internal bleaching (4 weeks); transplantation after orthodontic treatment completed
**Posttransplantation follow-up**		
4 weeks	Asymptomatic; EPT+.	Asymptomatic; EPT−
12 weeks	Asymptomatic; EPT+.	Asymptomatic; EPT+ and cold test+
24 weeks	EPT+; extruded radiopacity migrating into canal.	EPT+ and cold test+; progressive intracanal radiopacity
48 weeks	EPT+ (weakening); resolution of apical lesion and apical mineralization on CBCT.	EPT+ and cold test+; lesion regression with mineralization at perforation and apex on CBCT
96 weeks	EPT+ (further diminished); changes more pronounced; no change in midroot dentin thickness.	EPT+ and cold test+; near‐complete closure of perforation and further apical mineralization; selective mineralization at perforation side on 3D reconstruction
**Clinical examinations at 96-week follow-up**		
Percussion	None.	None
Palpation	None.	None
Mobility (Miller class)	Class 0.	Class 0
Probing depth (mm)	≦ 3.	≦ 3
**Quantitative radiographic assessment**		
Periapical lesion size at baseline (mm × mm)	3.0 × 1.0.	2.0 × 1.0
Periapical lesion size at 48 weeks (mm × mm)	Not detectable.	1.7 × 0.4
Periapical lesion size at 96 weeks (mm × mm)	Not detectable.	1.3 × 0.3
Perforation size at baseline (mm)	Not applicable^a^.	0.3
Perforation size at 48 weeks (mm)	Not applicable^a^.	0.2
Perforation size at 96 weeks (mm)	Not applicable^a^.	≤ 0.1
CBCT‐PAI score at baseline^b^	3D.	3D
CBCT‐PAI score at 48 weeks^b^	0.	2
CBCT‐PAI score at 96 weeks^b^	0.	1
Perforation defect volume immediately post‐Tx (mm^3^)^c^	Not applicable^a^.	0.1817
Perforation defect volume at 96 weeks (mm^3^)^c^	Not applicable^a^.	0.0769
Volume reduction at 96 weeks (%)	Not applicable^a^.	57.7
**Final outcome (96 weeks)**		
Tooth survival	Yes.	Yes
Symptoms	None.	None
Adverse events	None reported throughout 96‐week follow‐up.	None reported throughout 96‐week follow‐up

*Note:* Plus and minus signs (+/−) denote positive/negative responses.

Abbreviations: CBCT, cone‐beam computed tomography; DPSC, dental pulp stem cell; EPT, electric pulp testing; G‐CSF, granulocyte colony‐stimulating factor; MTA, mineral trioxide aggregate; PCR, polymerase chain reaction.

^a^Not applicable. The cervical perforation in Case 1 was sealed with mineral trioxide aggregate (MTA) prior to DPSC transplantation and was therefore not evaluable as a regenerative outcome; quantitative perforation measurements are reported only for Case 2.

^b^CBCT‐PAI score: cone‐beam computed tomography periapical index [11]. The size component is graded on a six‐point ordinal scale (0 = *intact periapical bone structure*; 1 = *radiolucency* 0.5–1 mm; 2 ≥ 1–2 mm; 3 ≥ 2–4 mm; 4 ≥4–8 mm; 5 ≥ 8 mm) and is supplemented when applicable by the modifiers E (expansion of the periapical cortical bone) and D (destruction of the periapical cortical bone). For example, “3D” denotes a periapical radiolucency of > 2–4 mm associated with destruction of the cortical bone.

^c^Volumetric analysis was performed by manual region‐of‐interest (ROI) segmentation of the residual nonmineralized space on serial CBCT slices using Horos, an open‐source DICOM viewer (Horos Project). The total volume was calculated as the sum of each ROI area multiplied by the slice thickness. Mean voxel grayscale values (Horos signal intensity) within the same volume of interest were 503.37 immediately posttransplantation and 508.75 at 96 weeks, confirming comparable image acquisition conditions between the two timepoints.

### 2.1. Case 1

**Figure 1:**
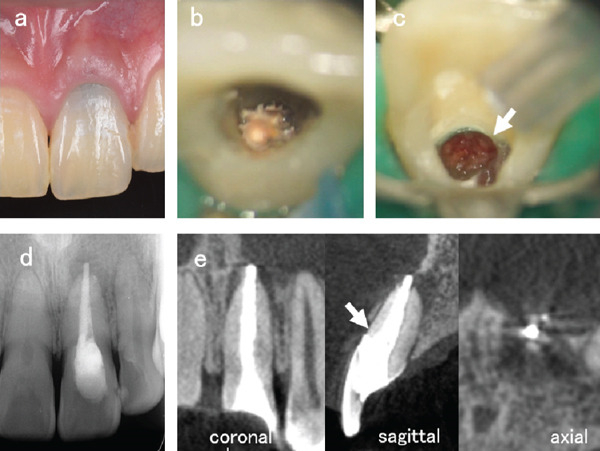
Case 1. Pretreatment clinical and radiographic findings of the maxillary left central incisor. (a) Frontal intraoral view at the initial visit showing discoloration of the cervical region of the affected tooth. (b) Microscopic view of the root canal orifice after access cavity preparation. (c) Cervical labial perforation (arrow) identified under the operating microscope. (d) Periapical radiograph at the initial visit showing root canal filling material extruded beyond the apex and a periapical radiolucency consistent with apical periodontitis. (e) Cone‐beam computed tomography (CBCT) images in coronal, sagittal, and axial views. The labial cervical perforation (arrow) and periapical bone resorption are clearly visualized. Clinically relevant findings: coexistence of cervical root perforation and apical periodontitis with extruded filling material, establishing the indication for regenerative cell therapy.

A 22‐year‐old male was referred to our clinic for RET of the left maxillary central incisor. The tooth had become devitalized due to a traumatic injury, was diagnosed with pulp necrosis, and had undergone endodontic treatment at another clinic when the patient was 15 years old. At the initial visit, the patient reported discomfort and tenderness in the periapical region, expressing concern about the discoloration of the cervical region of the affected tooth (Figure 1). Radiographs indicated root canal filling material protruding from the root apex, along with an apical lesion displaying periapical radiolucency. Further assessment using CBCT revealed bone resorption in the periapical region and perforation on the labial side of the cervical region. Baseline examination findings (percussion, palpation, probing depth, and mobility) are summarized in Table 2. The patient was thoroughly informed of these findings, along with the risks and benefits of RET using autologous DPSCs, and provided informed consent for the proposed therapy.

The maxillary left third molar was extracted and transported to a cell‐processing facility (Air Water Aeras Bio Inc., Kobe, Japan). Autologous DPSCs were isolated from the dental pulp by enzymatic digestion, cultured, and cryopreserved at the fourth passage following previously established protocols [12]. Cells used for transplantation were therefore at Passage 4. Prior to release for clinical use, each DPSC product was subjected to a comprehensive panel of quality and safety tests in accordance with the predefined release criteria approved by the Certified Committee for Regenerative Medicine. The release tests included (i) flow cytometric analysis of mesenchymal stem cell surface markers (CD29 and CD105 positive; CD31 negative) for identity; (ii) trypan blue exclusion assay for viability; (iii) measurement of population doubling time for proliferation capacity; (iv) sterility test for aerobic bacteria, anaerobic bacteria, and fungi for microbiological safety; (v) endotoxin quantification; and (vi) mycoplasma test. Only DPSC products meeting all of these release criteria were approved for transplantation. The numerical thresholds for each release test are proprietary to the cell‐processing facility; however, the actual values obtained for both cases are summarized in Table 2, and all results fully satisfied the predefined release criteria.

During the initial root canal treatment, gutta‐percha was removed using a gutta‐percha remover spear (YDM, Tokyo, Japan). After removal of the existing gutta‐percha, the cervical perforation site on the labial aspect of the root was identified and inspected under a surgical operating microscope. Inflammatory granulation tissue at the perforation site was carefully removed using a hand excavator. The site was then irrigated with sterile saline to ensure a clean surface. MTA (TMR MTA cement; Yamakin, Konan, Japan) was prepared according to the manufacturer′s instructions and applied directly into the perforation defect using an MTA carrier under microscopic visualization. The MTA was gently condensed against the external root surface using a hand plugger, and a moist cotton pellet was placed against the inner surface to allow setting. The setting of the MTA was confirmed at the next visit before proceeding with the subsequent root canal disinfection protocol.

At each visit, the root canal was disinfected using the following sequential protocol: (1) irrigation with 2.5% sodium hypochlorite (NaOCl) (2.5 mL, less than 2 min) using a 27‐G needle; (2) irrigation with EDTA (3% EDTA, SMEARCLEAN; Nippon Shika Yakuhin Co. Ltd., Shimonoseki, Japan; or 17% EDTA, 17% EDTA Liquid; Pentron Japan Inc., Tokyo, Japan; 2 mL, 1 min) using a 27G needle, with the concentration and exposure time selected according to the clinical purpose (e.g., apical patency, smear layer removal, or NaOCl neutralization); (3) rinsing with 5 mL of sterile saline using a 30G/31G needle; (4) irrigation with 2.5 mL of plain nanobubble water (Air Water Aeras Bio Inc., Kobe, Japan) using a 30G/31G needle, after which the irrigant was left in the canal for 2 min; and (5) irrigation with 2.5 mL of antibiotic‐containing nanobubble water using a 30G/31G needle, the remainder of which was left in the canal as the intracanal medication until the next visit (interval of 2–4 weeks). Detailed information on each step is provided in Table S1.

**Figure 2:**
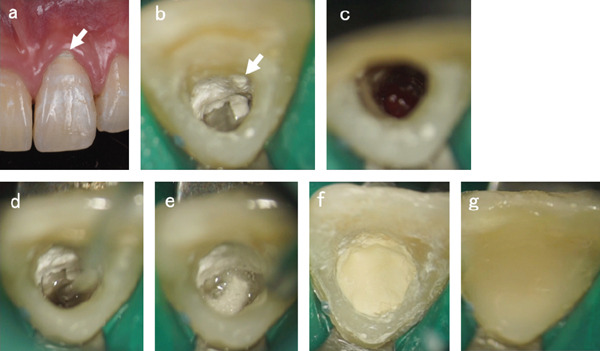
Case 1. Intraoperative clinical procedures during root canal disinfection and regenerative endodontic therapy (RET) using DPSCs. (a) Frontal view after orthograde retreatment showing gingival recession exposing the cervical region. (b, c) Microscopic views of the root canal after mechanical cleaning and perforation repair; the apical foramen is widely open. (d) Transplantation of the autologous DPSC suspension into the root canal using a syringe. (e) Placement of a gelatin sponge over the cell suspension at the level of the cervical perforation. (f) Sealing of the access cavity with Biodentine. (g) Final restoration with composite resin.

**Figure 3:**
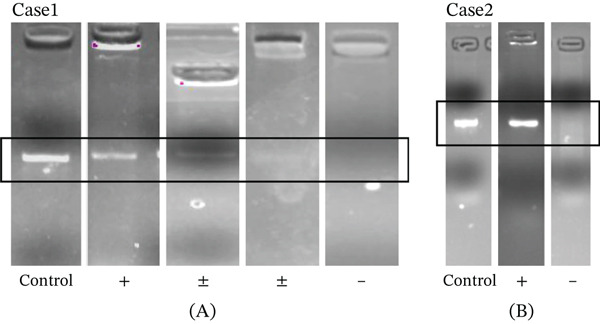
PCR‐based bacterial detection in root canal samples obtained before each treatment session. The boxed region indicates the bacterial 16S rDNA amplicon band. (A) Case 1: positive (+) at the first session, weakly positive (±) at the second and third sessions, and negative (−) at the fourth session, confirming progressive disinfection prior to cell transplantation. (B) Case 2: positive (+) at the first session and negative (−) at the second session. Clinically relevant finding: Bacterial DNA was confirmed to be below the PCR detection limit before cell transplantation in both cases, satisfying the microbiological prerequisite for the regenerative procedure.

Four weeks after the initial visit, the patient returned asymptomatic; however, the labial part of the MTA filling had become exposed to the oral microenvironment due to gingival recession (Figure 2a–c). At each visit, bacterial contamination was evaluated by PCR amplification of an approximately 0.8‐kb fragment of bacterial 16S rDNA, spanning the V1–V4 hypervariable regions, using the Bacterial 16S rDNA PCR Kit Fast (800) (Takara Bio Inc., Kusatsu, Japan), as described by Iohara et al. [3]. The PCR products were sequenced (commissioned to FASMAC Co. Ltd., Atsugi, Japan), and the bacterial species were identified using NCBI BLAST. Because bacterial DNA continued to be detected by PCR despite repeated disinfection, the antibiotic incorporated into the nanobubble water was empirically modified at each step based on PCR findings and sequencing‐identified bacterial species, with the aim of reducing bacterial DNA to below the detection limit before cell transplantation. Formal antimicrobial susceptibility testing was not performed; therefore, the antibiotic regimen was guided by PCR and sequencing results rather than by species‐specific susceptibility data. At the initial visit, levofloxacin (0.015%; Cravit Ophthalmic Solution, Santen Pharm, Co. Ltd., Osaka, Japan) was used as the empirical first‐line antibiotic. After sequencing identified *Streptococcus mitis* at the second visit, the antibiotic was empirically changed to doripenem (Finibax; Shionogi Pharma, Co. Ltd., Osaka, Japan) in an attempt to broaden antimicrobial coverage. Subsequent sequencing at the fourth visit identified *Lautropia mirabilis*, a gram‐negative coccobacillus for which formal antimicrobial susceptibility data in endodontic infections are limited; on this basis, the antibiotic was again empirically changed, this time to ampicillin (Viccillin; Meiji Seika Pharma Co. Ltd., Tokyo, Japan), with the aim of providing a different class of antimicrobial coverage. At the fifth visit, PCR analysis confirmed that bacterial DNA was below the detection limit. Although a pretransplantation radiograph revealed a granular opacity in the periapical region suggestive of remnants of a previous filling, no such material was observed inside the root canal under a surgical operating microscope. Cell transplantation was therefore performed subsequently (Figure 3).

Cryopreserved autologous DPSCs were transported from the cell‐processing center to the clinic. Immediately before cell transplantation, the root canal was irrigated with 17% EDTA (2.5 mL, 2 min) followed by sterile saline (5.0 mL). A cell suspension of 2 × 10^5^ cells containing granulocyte colony‐stimulating factor and atelocollagen was then transplanted, as previously reported [12]. A gelatin sponge (Spongel; LTL Pharma Inc., Tokyo, Japan) was applied to the cell suspension, filling the same position as that of the perforation near the root canal orifice. The tooth was then sealed using Biodentine (Septodont, Lancaster, Pennsylvania, United States) and composite resin (Figure 2d–g).

**Figure 4:**
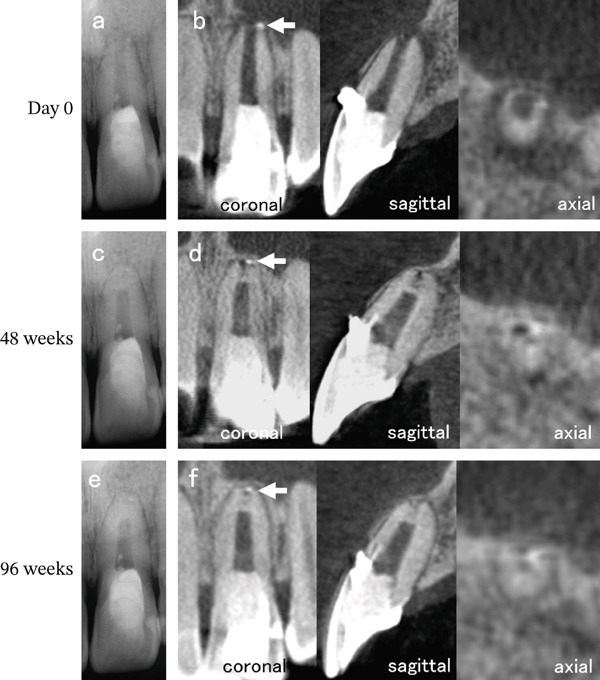
Case 1. Time‐course radiographic and CBCT evaluation of the maxillary left central incisor following DPSC transplantation. (a, c, e) Periapical radiographs and (b, d, f) CBCT images in coronal, sagittal, and axial views obtained immediately after transplantation (Day 0; a, b) and at (c, d) 48 and (e, f) 96 weeks postoperatively. Arrows indicate the periapical region containing the previously extruded radiopaque material. Day 0: Baseline status immediately after transplantation; periapical radiolucency and the radiopaque material extruded beyond the apex are visible. 48 weeks: resolution of the periapical radiolucency, apical mineralization within the root canal, and apparent inward displacement of the extruded radiopaque material into the canal space. 96 weeks: Further progression of apical mineralization with sustained absence of periapical radiolucency. Clinically relevant findings: progressive healing of apical periodontitis and intracanal mineralized tissue formation, consistent with favorable healing following the regenerative procedure.

The patient underwent follow‐up examinations at 1, 4, 12, 24, 48, and 96 weeks after the cell transplantation. Throughout this period, no subjective symptoms and no local or systemic adverse events related to the affected tooth were observed. The tooth exhibited a positive response to EPT after 4 weeks. Radiographs showed a gradual decrease in the size of the periapical opacity, with the residual opacity appearing to shift toward the root canal over time (Figure 4). Furthermore, CBCT after 48 weeks showed the disappearance of the apical lesion, and significant mineralization was observed in the root canal near the apex. At 96 weeks, these trends became more pronounced; however, there was no change in dentin thickness in the middle third of the root canal. The response to EPT gradually diminished after 48 weeks of follow‐up.

### 2.2. Case 2

**Figure 5:**
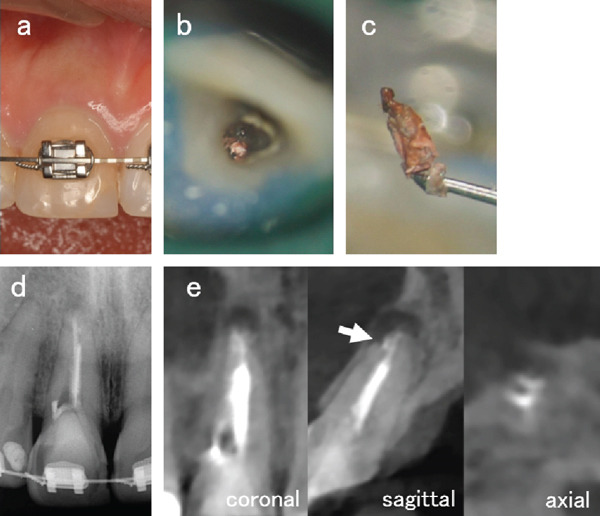
Case 2. Pretreatment clinical and radiographic findings of the maxillary right central incisor. (a) Frontal intraoral view at the initial visit during orthodontic treatment, showing discoloration of the affected tooth. (b) Microscopic view of the root canal orifice. (c) Removed root canal filling material. (d) Periapical radiograph showing sparse root canal filling and periapical radiolucency. (e) CBCT images (coronal, sagittal, and axial views). A perforation near the root apex (arrow) and an open apical foramen are visible. Clinically relevant findings: refractory apical periodontitis with an apical perforation, indicating the need for a regenerative cell‐based approach that targets both the root canal and the perforation defect.

A 28‐year‐old female was referred to our clinic for RET of the right maxillary central incisor. The tooth had undergone caries treatment at the age of 13 years but later developed spontaneous pain, leading to pulp removal at another clinic. Apical tenderness had been present since her early 20s, and the tenderness persisted despite undergoing root canal treatment for apical periodontitis at a different clinic. At the initial visit, the patient was undergoing orthodontic treatment, and the right maxillary central incisor displayed discoloration (Figure 5). Radiography revealed sparse root canal filling. CBCT images showed an apical lesion and a perforation near the apex. Baseline clinical findings are summarized in Table 2. After obtaining informed consent for cell therapy, autologous DPSCs were isolated from the left maxillary third molar (Table 1).

The existing root canal filling material appeared poorly condensed and partially degraded, suggesting possible microbial contamination of the canal; infection was subsequently confirmed by PCR analysis of intracanal samples before cell transplantation. Root canal preparation and disinfection were performed using the same sequential protocol as in Case 1 (i.e., the same concentrations, volumes, exposure times, and needle gauges). The same PCR‐based microbial monitoring was also applied; however, sequencing did not identify any pathogenic bacteria warranting a change from levofloxacin (0.015%), which was therefore used as the sole antibiotic in the nanobubble‐containing irrigant and intracanal medication throughout the treatment course. After removal of the previous root canal filling, the apical perforation site was identified under a surgical operating microscope. Residual gutta‐percha at the perforation area was carefully removed using a gutta‐percha spear remover (YDM, Tokyo, Japan), and any inflammatory tissue was gently debrided. Importantly, the perforation site was not sealed with MTA or other conventional repair materials prior to cell transplantation, but was instead treated directly with autologous DPSCs concurrently with the cell transplantation procedure. After root canal preparation, the upper part of the root canal was adequately sealed with hydraulic cement, and the cavity above the root canal orifice was filled with sodium perborate for 4 weeks to treat the discoloration. PCR analysis confirmed that bacterial DNA was below the detection limit before cell transplantation. RET using DPSCs was performed after orthodontic treatment was completed.

**Figure 6:**
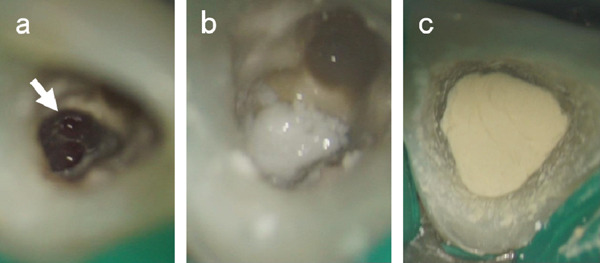
Case 2. Intraoperative clinical procedures of regenerative endodontic therapy (RET) using DPSCs. (a) Microscopic view showing the apical perforation and associated periapical lesion (arrow). (b) Placement of collagen‐based hydroxyapatite (ReFit Dental; HOYA Technosurgical Corp., Tokyo, Japan) over the root canal orifice following transplantation of the DPSC suspension into the canal and the perforation site. (c) Sealing with Biodentine and final restoration with composite resin.

Following completion of root canal disinfection and confirmation that bacterial DNA was below the PCR detection limit, the autologous DPSC suspension containing granulocyte colony‐stimulating factor and atelocollagen was delivered through the root canal orifice using a syringe, allowing the cell suspension to fill both the entire root canal lumen and the apical perforation defect under microscopic visualization. A collagen‐based hydroxyapatite scaffold, ReFit Dental (HOYA Technosurgical Corp., Tokyo, Japan), was then placed over the cell suspension at the level of the root canal orifice to retain the cell suspension within the root canal. The access cavity was finally sealed with Biodentine followed by composite resin (Figure 6). This case was characterized by direct transplantation of autologous DPSCs into the perforation defect without prior sealing with conventional repair materials.

**Figure 7:**
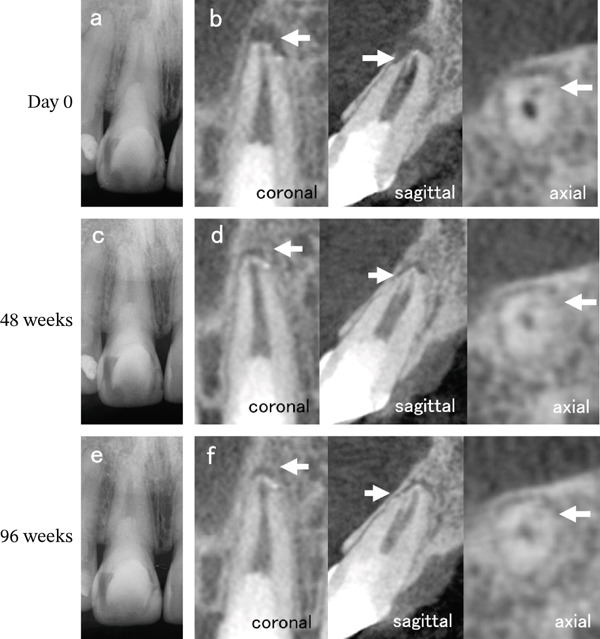
Case 2. Time‐course radiographic and CBCT evaluation of the maxillary right central incisor following DPSC transplantation. (a, c, e) Periapical radiographs and (b, d, f) CBCT images in coronal, sagittal, and axial views obtained immediately after transplantation (Day 0; a, b) and at (c, d) 48 and (e, f) 96 weeks postoperatively. Arrows indicate the perforation site near the root apex. Day 0: Open apical perforation and periapical radiolucency are visible. 48 weeks: increasing radiopacity within the root canal, near‐complete sealing of the apical perforation by hard tissue, and regression of the periapical lesion. 96 weeks: further consolidation of the mineralized tissue at the perforation site and continued resolution of the periapical lesion. Clinically relevant findings: selective hard‐tissue formation at the perforation site and progressive healing of the periapical lesion without the use of conventional perforation repair materials.

**Figure 8:**
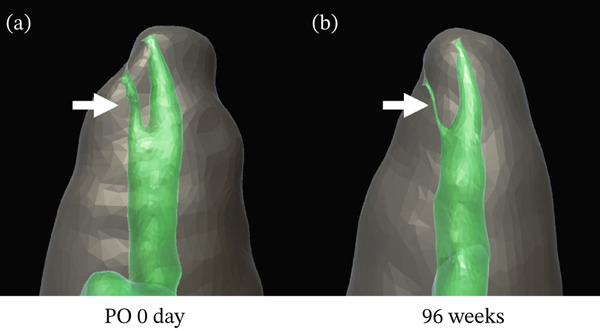
Case 2. Three‐dimensional reconstructed CBCT images of the apical region. (a) Immediately after cell transplantation (postoperative day 0). (b) At 96 weeks postoperatively. Arrows indicate the perforation site on the labial aspect of the apex. Clinically relevant finding: progressive narrowing and mineralization localized to the perforation site, whereas the original apical foramen remains relatively unchanged. This pattern suggests localized mineralized tissue formation at the perforation site rather than diffuse apical closure.

The tooth exhibited positive responses to both EPT and cold tests first detected after 12 weeks, and these responses persisted even after 48 weeks. Radiography and CBCT imaging indicated a tendency for remission of the apical lesion after 48 weeks (Figure 7). Furthermore, CBCT imaging through sagittal and axial views after 48 weeks revealed mineralized tissue formation in the perforation and apical sites of the root canal compared with the images obtained immediately after cell transplantation. No pain, swelling, or functional instability of the tooth was observed, and no local or systemic adverse events occurred during the follow‐up period. At 96 weeks, these trends were more pronounced (Figure 7a–f). Furthermore, a comparison of 3D images of the apical area immediately after transplantation and at 96 weeks (DenPre 3D Lab, Dental Prediction, Tokyo, Japan) revealed that one side was more mineralized, suggesting that the root perforation site underwent localized mineralization in parallel with the regenerative endodontic treatment (Figure 8a,b). This spatial and temporal association does not, by itself, establish that the transplanted cells directly repaired the perforation. To quantitatively assess hard‐tissue formation at the perforation site, three‐dimensional volumetric analysis of CBCT images was performed using Horos, an open‐source DICOM viewer (Horos Project; https://horosproject.org/). Measurements were obtained at two timepoints: immediately after DPSC transplantation and at 96 weeks postoperatively. On every CBCT slice containing the perforation defect, the boundary of the residual nonmineralized space was manually delineated as a region of interest (ROI), and the total volume (mm^3^) was calculated as the sum of each ROI area multiplied by the slice thickness (0.15 mm). The mean voxel grayscale value within the same region (referred to as signal intensity in Horos) was also recorded at each timepoint as an internal quality‐control measure to verify the comparability of the two image sets. The volume of the residual nonmineralized space at the perforation defect decreased from 0.1817 mm^3^ immediately after transplantation to 0.0769 mm^3^ at 96 weeks, corresponding to a 57.7% reduction. Over the same period, the mean grayscale value remained essentially unchanged (503.37 vs. 508.75), supporting the validity of the intertime‐point volumetric comparison.

At the final follow‐up, both patients reported no subjective tooth‐related symptoms and expressed satisfaction with the regenerative endodontic treatment itself. The patient in Case 1 noted residual dissatisfaction with gingival recession at the labial cervical area, and the patient in Case 2 reported slight dissatisfaction with residual mild tooth discoloration; neither concern was attributable to the cell transplantation procedure.

## 3. Discussion

This report investigated the feasibility of RET using autologous DPSCs in mature teeth with apical periodontitis complicated by root perforation. Cell‐free regenerative endodontic procedures can achieve favorable clinical and radiographic outcomes [1], but the resulting tissue is generally repair tissue (periodontal ligament‐, cementum‐, or bone‐like) rather than a true regenerated dentin–pulp complex [2]. The rationale for using autologous DPSCs in the present cases was therefore to create a biological environment in which living tissue could repopulate the root canal and, in Case 2, the perforation site itself.

Root perforation creates a direct communication between the infected root canal and the surrounding periodontal tissues, and persistent leakage can compromise tooth survival [6]. MTA and other calcium silicate–based materials are established repair materials because of their sealing ability and biocompatibility [6–9]. In Case 1, the cervical perforation was sealed with MTA before cell transplantation, following the principle that leakage control is a prerequisite for regeneration; the favorable clinical and radiographic course suggests that autologous DPSC transplantation is compatible with an MTA‐based presealing strategy. A noteworthy observation is provided by Case 2, in which the perforation was near the root apex and continuous with the periapical lesion, making conventional repair more difficult than for a cervical perforation. Instead of presealing the defect, the autologous DPSC suspension was transplanted directly into both the root canal and the perforation site. To the author′s knowledge, this is the first clinical description of such an approach for an apical perforation in a mature tooth with apical periodontitis, treating the perforation as a biological space into which regenerative cells were introduced with the aim of promoting mineralized, tissue‐integrated repair. This represents an exploratory clinical approach, and any contribution of the transplanted cells to the observed repair should be interpreted as an association rather than as a demonstrated causal effect.

The biological rationale is supported by peer‐reviewed evidence. In a dog model of mature teeth with apical periodontitis, autologous DPSC transplantation after strict periapical disinfection histologically supported regeneration of pulp‐like tissue with adjacent dentin‐like mineralized tissue [3]. Preclinical studies have also suggested that DPSCs and DPSC‐derived conditioned medium contribute to periodontal ligament regeneration through trophic effects, including pulp–periodontal cross talk mediated by VEGF and HGF [13] and periostin‐dependent regulation of the host microenvironment [14]. Clinically, autologous DPSC transplantation in mature molars has been reported as feasible with favorable outcomes [12], and regenerative endodontic treatment has also been reported for perforating root resorption, although that scenario differs in tooth maturity, pathology, and etiology [15]. Collectively, these findings suggest that DPSC‐based therapy may engage the broader pulp–periodontal complex through both cellular contribution and paracrine signaling, although none fully replicates the scenario of Case 2; the present approach should therefore be regarded as an exploratory extension rather than a validated protocol. Histological characterization of the tissue formed at the perforation site remains an essential next step.

Strict infection control was essential. A marked reduction in bacterial load has been suggested to create a root canal environment suitable for pulp regeneration [16], and residual bacteria including *Enterococcus faecalis* may impair pulp regeneration in animal models [3, 17]. In the present cases, PCR analysis monitored intracanal contamination, and cell transplantation was performed only after bacterial DNA was undetectable. In Case 1, *S*. *mitis* and *L*. *mirabilis* were detected during treatment, and the antibiotic regimen was empirically adjusted at each step, although formal susceptibility‐based selection was not performed. Microbiological confirmation was particularly important in Case 2, where direct placement of DPSCs into an unsealed apical perforation would be unlikely to succeed if the defect remained an active pathway for infection.

In Case 1, EPT became positive 4 weeks after transplantation, and by 48 weeks CBCT showed resolution of the apical lesion with mineralized tissue formation in the apical part of the canal; these changes were more pronounced at 96 weeks. The subsequent weakening of the EPT response after 48 weeks may be explained by two principal, nonmutually‐exclusive sets of factors that the present uncontrolled observation cannot distinguish. (i) Biological factors within the newly formed tissue: Progressive mineralized tissue formation may have restricted nerve fiber extension, and/or the newly formed tissue itself may have undergone remodeling during its maturation phase, with transient changes in neural density or fiber organization. (ii) Methodological factors related to EPT measurement: inherent sampling variability between visits, including differences in tooth surface moisture, conductive medium, and electrode contact area, together with attenuation of the stimulus current by the overlying Biodentine and composite resin restoration. Without histological verification or systematic measurement controls, the originally proposed mineralization‐based mechanism should therefore be regarded as one possible explanation rather than the established cause. In Case 2, both EPT and cold test responses became positive after 12 weeks and persisted at 48 weeks; this broader sensory response is clinically meaningful, as cold responsiveness may indicate recovery of sensory components not fully assessed by EPT alone.

Quantitative radiographic outcomes were also favorable in both cases. At baseline, both teeth were graded as CBCT‐PAI 3D, indicating periapical radiolucencies of > 2–4 mm associated with cortical bone destruction. By 96 weeks, the cortical bone destruction modifier had resolved in both cases (CBCT‐PAI 0 in Case 1; CBCT‐PAI 1 in Case 2), suggesting reconstitution of the periapical cortical plate concurrent with size reduction of the radiolucency. The radiographic pattern in Case 2 is particularly noteworthy. At 48 weeks, imaging showed remission of the apical lesion and mineralized tissue formation at both the apical and perforation sites; by 96 weeks, three‐dimensional reconstruction suggested selective mineralization at the perforation defect into which the DPSC suspension had been directly transplanted.

This spatial correspondence between DPSC delivery and hard‐tissue deposition supports the plausibility that the transplanted cells contributed to perforation repair, although radiographic closure cannot identify the tissue type (dentin‐like, cementum‐like, bone‐like, or mixed). Direct evidence for cementum regeneration by DPSCs alone in this clinical setting remains limited. Even so, near‐complete closure of an apical perforation without MTA presealing suggests that DPSC‐based therapy may contribute to hard‐tissue formation at the perforation interface, rather than only supporting periapical healing at a distance. These observations complement a previous report of allogeneic umbilical cord–derived mesenchymal stem cell therapy in a mature tooth with apical periodontitis and cervical root perforation, in which the perforation was sealed before cell implantation [10]. The present Case 1 follows a similar preseal concept using autologous DPSCs, whereas Case 2 extends it by including the perforation defect within the transplantation field. Thus, autologous DPSCs may serve two clinical roles in teeth with perforation: as an intracanal cell–based regenerative treatment following conventional perforation sealing, and as a direct cell‐based therapy targeting the perforation defect itself when conventional sealing is not feasible. The biological process in both settings involves the same transplanted cells; the distinction is one of anatomic target rather than mechanism. An adequate coronal seal was provided in both cases by Biodentine beneath composite resin [18, 19]. In Case 1, Biodentine was placed over a gelatin sponge, whereas in Case 2, it was placed over a collagen‐based hydroxyapatite scaffold. No clinical or radiographic signs of leakage or treatment failure were observed during 96 weeks of follow‐up, and no local or systemic adverse events occurred, supporting the preliminary safety of the protocol.

Several limitations of this report should be acknowledged. First, this report describes only two uncontrolled cases treated by a single operator at a single center, with no comparator group treated by conventional MTA‐based perforation repair, orthograde retreatment, surgical endodontic intervention, or extraction. Selection bias inherent to clinical case reports cannot be excluded, and the findings therefore demonstrate feasibility in carefully selected patients but cannot establish therapeutic efficacy or support quantitative comparison with established perforation repair materials. Second, no histological confirmation was possible, because such evaluation would have required extraction of asymptomatic functional teeth and was not ethically justifiable. The radiopaque material observed on CBCT within the canal and at the perforation interface may represent dentin‐like tissue produced by transplanted DPSCs, cementum‐ or bone‐like tissue derived from host cells recruited from the periapical region, or mixed mineralized repair tissue similar to that previously described after cell‐free regenerative endodontic procedures [2]. The present findings therefore cannot distinguish true regeneration of the pulp–dentin complex, as suggested by preclinical studies [3, 12], from reparative mineralized tissue. Accordingly, the term “regenerative endodontic therapy using DPSCs” should be understood as describing the intent and biological rationale of the treatment, not as a histologically verified outcome. Third, all outcome measures used in this report are indirect. EPT and cold testing assess sensory responsiveness but do not identify the tissue type that produces the response, whereas CBCT, periapical radiography, and the CBCT‐PAI quantify radiographic healing without distinguishing regenerated pulp‐like tissue from periapical bone repair achieved by infection control alone. Direct measures of vascularity (e.g., laser Doppler flowmetry, and pulse oximetry) and innervation were not performed and should be incorporated into future investigations where ethically and technically feasible. Fourth, although 96 weeks is comparatively long for a clinical case report, it remains short relative to the expected functional service of anterior teeth in young adults; the long‐term stability of the mineralized tissue, the risk of late reinfection or root resorption, and the durability of the coronal seal cannot be assessed within this observation period. Fifth, both cases involved maxillary central incisors in young patients aged 22 and 28 years, so the findings cannot be extrapolated to molars, periodontally compromised teeth, older patients, or different perforation locations or sizes; in addition, the protocol was delivered within Japan′s Class II regenerative medicine framework using a contracted cell‐processing facility with proprietary release criteria, and certain operational aspects may not be directly transferable to other regulatory or institutional settings. Sixth, the transplanted cells were coadministered with several biological components—granulocyte colony‐stimulating factor and atelocollagen in both cases, a gelatin sponge in Case 1, and a collagen‐based hydroxyapatite scaffold in Case 2—and the individual contribution of each component was not evaluated separately. Consequently, the mineralized tissue observed within the root canal and at the perforation interface cannot be attributed solely to the transplanted DPSCs, nor to the atelocollagen, granulocyte colony‐stimulating factor, gelatin sponge, or scaffold material individually; their respective and combined roles remain to be defined in controlled studies.

Within these limitations, the present report demonstrates the clinical feasibility of autologous DPSC transplantation and its favorable safety profile over a 96‐week follow‐up period in previously endodontically treated mature anterior teeth with apical periodontitis and root perforation when performed in young patients under strict infection control and within an appropriate regulatory framework. The favorable clinical, radiographic, and sensory outcomes observed over this period suggest that this approach may offer a biologically based treatment option for selected cases in which conventional management is challenging. Although these findings do not establish superiority over MTA‐based perforation repair or provide histological proof of pulp–dentin complex regeneration, they provide a useful basis for further investigation. Larger comparative studies with longer follow‐up, validated surrogate biological markers, and histological validation where ethically feasible will help clarify the therapeutic potential, durability, and appropriate indications of this approach. Taken together, the present findings support a biologically plausible, tooth‐preserving approach, but they do not constitute definitive proof of pulp–dentin complex regeneration.

## 4. Conclusions

In these two cases of mature maxillary incisors with persistent apical periodontitis and root perforation, transplantation of autologous DPSCs with G‐CSF and atelocollagen was feasible and was associated with favorable clinical and radiographic outcomes over 96 weeks of follow‐up, including remission of periapical lesions, mineralized tissue formation in the apical region, and near‐complete closure of the perforation site in Case 2. No adverse events were observed. Because of the uncontrolled design, the very small sample size, and the absence of histological confirmation, these findings should be regarded as preliminary evidence of feasibility rather than as proof of pulp–dentin complex regeneration, and they do not establish superiority over established perforation repair materials such as MTA. Accordingly, this report should be interpreted as preliminary evidence of feasibility, safety, and biological promise; it does not validate autologous DPSC transplantation for routine clinical use, which will require confirmation in larger, controlled studies with longer follow‐up and, where ethically feasible, histological validation.

## Author Contributions

**Ryosuke Matsuki** was responsible for all aspects of this work, including conceptualization, methodology, investigation, resources, data curation, formal analysis, validation, visualization, original draft preparation, manuscript review and editing, supervision, and project administration. This included performing the clinical treatment, collecting, curating, analyzing, and interpreting the clinical, radiographic, and follow‐up data, preparing the figures and tables, and drafting, revising, and approving the final version of the manuscript. Cell processing was outsourced to Air Water Aeras Bio Inc. under the accepted regenerative medicine provision plan and was not performed by the author.

## Funding

No funding was received for this manuscript.

## Disclosure

The author has read and approved the final version of the manuscript. The corresponding author and manuscript guarantor had full access to all of the data in this study and takes complete responsibility for the integrity of the data and the accuracy of the data analysis. The cell‐processing contractor had no role in the design of this case report, in data collection, in data analysis or interpretation, in manuscript preparation, or in the decision to submit the manuscript for publication. Dr. Nakashima had no role in patient selection, clinical treatment, data collection, data analysis, manuscript preparation, or the decision to submit.

## Conflicts of Interest

Cell processing for autologous DPSC transplantation was outsourced to Air Water Aeras Bio Inc. (Kobe, Japan), a subsidiary of Air Water Inc., under the accepted regenerative medicine provision plan. Dr. Misako Nakashima, acknowledged for providing technical instruction on the RET using DPSCs, served as a founding director of Aeras Bio (the original entity now operating as Air Water Aeras Bio Inc.) and retired from that directorship approximately 5 years before manuscript preparation. Since November 2025, she has served as a special advisor to the Regenerative Medicine Research Institute of Air Water Inc., the parent company of the cell‐processing contractor; this affiliation is disclosed here for transparency. She is currently affiliated with the Future Health Medical Corporation, RD Dental Clinic, Japan, which is independent of the cell‐processing contractor. The author declares no other conflicts of interest.

## Supporting information


**Supporting Information** Additional supporting information can be found online in the Supporting Information section. Table S1: The detailed irrigation and intracanal medication protocol used during root canal disinfection before autologous DPSC transplantation in both cases.

## Data Availability

The data that support the findings of this study are available from the corresponding author upon reasonable request.

## References

[1] Youssef A. , Ali M. , ElBolok A. , and Hassan R. , Regenerative Endodontic Procedures for the Treatment of Necrotic Mature Teeth: A Preliminary Randomized Clinical Trial, International Endodontic Journal. (2022) 55, no. 4, 334–346, 10.1111/iej.13681, 35030270. 35030270

[2] Lin L. M. , Huang G. T. , Sigurdsson A. , and Kahler B. , Clinical Cell-Based Versus Cell-Free Regenerative Endodontics: Clarification of Concept and Term, International Endodontic Journal. (2021) 54, no. 6, 887–901, 10.1111/iej.13471, 33389773. 33389773

[3] Iohara K. , Tominaga M. , Watanabe H. , and Nakashima M. , Periapical Bacterial Disinfection Is Critical for Dental Pulp Regenerative Cell Therapy in Apical Periodontitis in Dogs, Stem Cell Research & Therapy. (2024) 15, no. 1, 10.1186/s13287-023-03628-6, 38229184. PMC10792888 38229184

[4] Brizuela C. , Meza G. , Urrejola D. , Quezada M. A. , Concha G. , Ramírez V. , Angelopoulos I. , Cadiz M. I. , Tapia-Limonchi R. , and Khoury M. , Cell-Based Regenerative Endodontics for Treatment of Periapical Lesions: A Randomized, Controlled Phase I/II Clinical Trial, Journal of Dental Research. (2020) 99, no. 5, 523–529, 10.1177/0022034520913242, 32202965. 32202965

[5] Nakashima M. and Tanaka H. , Pulp Regenerative Therapy Using Autologous Dental Pulp Stem Cells in a Mature Tooth With Apical Periodontitis: A Case Report, Journal of Endodontics. (2024) 50, no. 2, 189–195, 10.1016/j.joen.2023.10.015, 37923123. 37923123

[6] Küçükkaya E. S. , Clinical Applications of calciumsilicate‐basedmaterials: A Narrative Review, Australian Dental Journal. (2023) 68, no. S1, S96–S109, 10.1111/adj.12986. 37885314

[7] Katsamakis S. , Slot D. E. , Van der Sluis L. W. M. , and Van der Weijden F. , Histological Responses of the Periodontium to MTA: A Systematic Review, Journal of Clinical Periodontology. (2013) 40, no. 4, 334–344, 10.1111/jcpe.12058, 23405962. 23405962

[8] Gorni F. G. , Andreano A. , Ambrogi F. , Brambilla E. , and Gagliani M. , Patient and Clinical Characteristics Associated With Primary Healing of Iatrogenic Perforations After Root Canal Treatment: Results of a Long-Term Italian Study, Journal of Endodontics. (2016) 42, no. 2, 211–215, 10.1016/j.joen.2015.11.006, 26743731. 26743731

[9] Wang X. , Xiao Y. , Song W. , Ye L. , Yang C. , Xing Y. , and Yuan Z. , Clinical Application of Calcium Silicate-Based Bioceramics in Endodontics, Journal of Translational Medicine. (2023) 21, no. 1, 10.1186/s12967-023-04550-4, 38007432. PMC10676601 38007432

[10] Cordero C. B. , Santander G. M. , González D. U. , Quezada A. , Silva C. I. , Vásquez C. , Jara R. , Jara D. , and Khoury M. , Allogeneic Cellular Therapy in a Mature Tooth With Apical Periodontitis and Accidental Root Perforation: A Case Report, Journal of Endodontics. (2020) 46, no. 12, 1920–1927.e1, 10.1016/j.joen.2020.04.007, 32532626. 32532626

[11] Estrela C. , Bueno M. R. , Azevedo B. C. , Azevedo J. R. , and Pécora J. D. , A New Periapical Index Based on Cone Beam Computed Tomography, Journal of Endodontics. (2008) 34, no. 11, 1325–1331, 10.1016/j.joen.2008.08.013, 18928840. 18928840

[12] Nakashima M. , Fukuyama F. , and Iohara K. , Pulp Regenerative Cell Therapy for Mature Molars: A Report of 2 Cases, Journal of Endodontics. (2022) 48, no. 10, 1334–1340.e1, 10.1016/j.joen.2022.07.010, 35940319. 35940319

[13] Futenma T. , Hayashi Y. , Iida N. , Nakamura K. , Sakatoku S. , and Nawa H. , Interaction of Pulp and Periodontal Ligament in Treatment of Trauma, Journal of Hard Tissue Biology. (2023) 32, no. 4, 231–238, 10.2485/jhtb.32.231.

[14] Sakatoku S. , Hayashi Y. , Futenma T. , Sugita Y. , Ishizaka R. , Nawa H. , and Iohara K. , Periostin Is a Candidate Regulator of the Host Microenvironment in Regeneration of Pulp and Dentin Complex and Periodontal Ligament in Transplantation With Stem Cell-Conditioned Medium, Stem Cells International. (2024) 2024, no. 1, 7685280, 10.1155/2024/7685280, 38435089. 38435089 PMC10907099

[15] Kaval M. E. , Güneri P. , and Çalışkan M. K. , Regenerative Endodontic Treatment of Perforated Internal Root Resorption: A Case Report, International endodontic journal. (2018) 51, no. 1, 128–137, 10.1111/iej.12784, 28439906. 28439906

[16] Kim S. G. , Infection and Pulp Regeneration, Dentistry Journal. (2016) 4, no. 1, 10.3390/dj4010004, 29563446. PMC5851207 29563446

[17] Verma P. , Nosrat A. , Kim J. R. , Price J. B. , Wang P. , Bair E. , Xu H. H. , and Fouad A. F. , Effect of Residual Bacteria on the Outcome of Pulp Regeneration In Vivo, Journal of Dental Research. (2017) 96, no. 1, 100–106, 10.1177/0022034516671499, 27694153. 27694153

[18] Samuel A. , Asokan S. , Geetha Priya P. R. , and Thomas S. , Evaluation of Sealing Ability of Biodentine and Mineral Trioxide Aggregate in Primary Molars Using Scanning Electron Microscope: A Randomized Controlled In Vitro Trial, Contemporary Clinical Dentistry. (2016) 7, no. 3, 322–325, 10.4103/0976-237X.188547, 27630495. 27630495 PMC5004544

[19] Tang J. J. , Shen Z. S. , Qin W. , and Lin Z. , A Comparison of the Sealing Abilities Between Biodentine and MTA as Root-End Filling Materials and Their Effects on Bone Healing in Dogs After Periradicular Surgery, Journal of Applied Oral Science. (2019) 27, e20180693, 10.1590/1678-7757-2018-0693, 31596370. 31596370 PMC6768120

