# Supplementary material for: Autologous Dental Pulp Stem Cell Transplantation for Mature Teeth With Apical Periodontitis and Root Perforation: Clinical and Radiographic Outcomes in Two Cases
Source: Case Rep Dent. 2026 Jul 10;2026:1311942. doi: 10.1155/crid/1311942 (PMC13352355; doi:10.1155/crid/1311942)
Supplement: Supplementary file 1 — Supporting Information Additional supporting information can be found online in the Supporting Information section. Table S1: The detailed irrigation and intracanal medication protocol used during root canal disinfection before autologous DPSC transplantation in both cases. [file CRID-2026-1311942-s001.docx]

**Supplementary Table 1.** Detailed irrigation and intracanal medication protocol applied at each treatment visit.

| **Step** | **Agent / Solution** | **Concentration** | **Volume** | **Exposure time** | **Needle gauge** |
| --- | --- | --- | --- | --- | --- |
| 1 | Sodium hypochlorite (NaOCl) | 2.5% | 2.5 mL | < 2 min | 27G |
| 2 | EDTA (SMEARCLEAN®, Nippon Shika Yakuhin Co., Ltd., Shimonoseki, Japan; or 17% EDTA Liquid, Pentron Japan Inc., Tokyo, Japan) | 3% or 17%† | 2.0 mL | Variable† | 27G |
| 3 | Sterile saline | — | 5.0 mL | Irrigation | 30G / 31G |
| 4 | Plain nanobubble water  (Air Water Aeras Bio Inc., Kobe, Japan) | — | 2.5 mL | Left in canal  for 2 min | 30G / 31G |
| 5 | Antibiotic-containing nanobubble water* | See footnote* | 2.5 mL | Irrigation + intracanal medication until the next visit ‡ | 30G / 31G |

**Note:** This irrigation sequence (Steps 1–5) was applied at every treatment visit in both cases. The interval between visits was 2–4 weeks. Visits continued until PCR analysis demonstrated bacterial DNA below the detection limit, after which autologous DPSC transplantation was performed. Immediately before cell transplantation, the protocol was modified to a single final irrigation with 17% EDTA (2.5 mL, 2 min) followed by sterile saline (5.0 mL).

**Footnotes:**

† EDTA concentration and exposure time were selected according to the clinical purpose at each visit (e.g., apical patency, smear layer removal, or neutralization of NaOCl). Immediately before cell transplantation, 17% EDTA (2 mL, 2 min) was applied as the standardized final irrigation step.

*Antibiotic incorporated into the nanobubble water:

**Case 1:** 0.015% levofloxacin (Cravit Ophthalmic Solution, Santen Pharmaceutical, Osaka, Japan) was used at the initial visit. Following PCR-based detection of *Streptococcus mitis*, the antibiotic was switched to doripenem (Finibax; Shionogi Pharma, Co., Ltd., Osaka, Japan) at a final concentration of 0.05% (0.5 mg/mL); following subsequent detection of *Lautropia mirabilis*, switched to ampicillin (Viccillin; Meiji Seika Pharma, Co., Ltd., Tokyo, Japan) at a final concentration of 0.25% (2.5 mg/mL). Cell transplantation was performed only after PCR analysis showed bacterial DNA below the detection limit.

**Case 2:** 0.015% levofloxacin was used as the sole antibiotic throughout the treatment course, as sequencing did not identify any pathogenic bacteria warranting a change.

‡ After irrigation, the antibiotic-containing nanobubble water remaining in the root canal was used as the intracanal medication and was renewed at each visit.

Abbreviations: DPSC, dental pulp stem cell; EDTA, ethylenediaminetetraacetic acid; PCR, polymerase chain reaction.
